# Pandemic play moderates the relation between caregiver stress and child emotional distress in contexts of economic adversity

**DOI:** 10.3389/fpsyg.2023.1155617

**Published:** 2023-06-02

**Authors:** María Fernanda Rueda-Posada, Rachel B. Thibodeau-Nielsen, Shannon E. Dier, Alaina Wilson-Dooley, Francisco Palermo, Rachel E. White, Christina Chung

**Affiliations:** ^1^Department of Human Development and Family Science, University of Missouri, Columbia, MO, United States; ^2^Department of Psychology, Hamilton College, Clinton, NY, United States

**Keywords:** COVID-19, child adjustment, pretend play, caregiver stress, resiliency

## Abstract

It is well-established that caregiver stress is linked to increased emotional distress among children, and recent evidence highlights similar associations between caregiver and child emotional well-being during the coronavirus (COVID-19) pandemic. Examining protective factors and coping mechanisms that are associated with resiliency in the face of pandemic-related stress can highlight potential strategies that may help children adapt to other unexpected hardships outside of a global pandemic. Previous research found that playing about the pandemic moderated an association between caregiver stress and children’s emotional distress. However, few studies have explored “pandemic play” among children from low-income households, where pandemic-related stressors were often exacerbated. In the present study, 72 caregivers of Head Start preschoolers between 3 and 6  years of age were surveyed between late 2020 and early 2021. Results revealed that 32% of children engaged in pandemic play frequently. Caregiver stress was positively associated with child emotional distress, but only among children who did not engage in pandemic play frequently. These findings support the idea that child-directed play may be a developmentally appropriate and accessible coping mechanism to reduce the emotional burden of stressful events on children, regardless of economic context.

## 1. Introduction

At the onset of the coronavirus (COVID-19) pandemic, there were many questions about its possible negative consequences for children and families’ well-being (Prime et al., 2020). Research has since demonstrated these concerns were well-founded. Longitudinal studies initiated before March 2020 captured considerable increases in parents’ negative moods (Gassman-Pines et al., 2020), caregivers’ depression (Pitchik et al., 2021), and children’s internalizing and externalizing behavior problems throughout the first year of the pandemic (Feinberg et al., 2022). Cross-sectional studies also demonstrated associations between pandemic-related stressors (e.g., positive COVID-19 cases, disruptions to children’s learning, family financial difficulties) and parent stress, anxiety, and depressive symptoms (e.g., Brown et al., 2020; Thibodeau-Nielsen et al., 2021). Furthermore, parents reported an increase in emotional lability and negative emotions for themselves and their children in qualitative studies (Weaver and Swank, 2021; Duran and Ömeroğlu, 2022).

The pandemic provided a rare opportunity to examine how caregivers and children were impacted by unexpected hardships and to identify the factors that facilitated their ability to cope with challenges. Play has been suggested as an important coping mechanism for children during times of uncertainty, such as the COVID-19 pandemic (e.g., Chatterjee, 2018; Cray, 2020; Pelly, 2020; Graber et al., 2021; Thibodeau-Nielsen et al., 2021). However, more work is needed to explore the potential protective role of play, especially for children facing economic adversity, where stress and uncertainty are often elevated (Kerr et al., 2021; Suarez-Lopez et al., 2021; Zheng et al., 2021). To that end, the present study examined the association between caregivers’ stress and their preschooler’s emotional distress within low-income families during the COVID-19 pandemic and whether this association varied depending on children’s engagement in play.

Family stress theories and research highlight prospective links between caregivers’ stress and children’s later emotional distress (Masarik and Conger, 2017). As such, pandemic-related increases in caregivers’ stress would be expected to increase children’s emotional distress. During the COVID-19 pandemic, parents across the world reported feeling overwhelmed by increased workloads and expressed difficulty handling their children’s negative emotions (Toran et al., 2021). Maternal psychological distress was associated with child behavior problems (Petrocchi et al., 2020; Shelleby et al., 2022), including heightened externalizing and internalizing behaviors (Hanetz-Gamliel et al., 2021). The association between pandemic-related stressors and children’s well-being was generally indirect, with caregivers’ stress identified as a mediating mechanism in multiple studies (e.g., Spinelli et al., 2020; Thibodeau-Nielsen et al., 2021). Collectively, such findings suggest a common path by which caregiver stress during the pandemic was related to children’s emotional well-being.

It is important to identify factors that can protect children’s emotional well-being during stressful situations, including the COVID-19 pandemic and beyond. Studies examining children’s coping in uncertain contexts, like hospitals and immigration detention centers, show that play is a key mechanism by which children can express themselves and process emotions (see Graber et al., 2021, for review). Pretend play, specifically, seems to be a coping strategy that young children naturally rely on, and research suggests that it can buffer the negative impact of stressful experiences at home (Russ, 2007; Thibodeau-Nielsen et al., 2020b). When engaging in pretend play, children have a controlled setting in which to recreate and process their lived experiences, allowing them to safely process their feelings (Russ, 2004; Russ and Fehr, 2016). It is therefore unsurprising that when asked about how their children managed stress during the pandemic, some parents reported their children engaged in physical activity and play, including pretend play (Tambling et al., 2021). Indeed, almost half of the parents surveyed by González et al. (2020) reported an increase in their children’s imaginative play, including pandemic-related play which occurred among approximately 20% of the children.

Playing about the pandemic, or pandemic play, includes behaviors such as pretending to diagnose a stuffed animal with COVID-19 or setting up an imaginary virtual birthday party (Cray, 2020; Pelly, 2020; Underwood, 2020; Thibodeau-Nielsen et al., 2021). Importantly, emerging research suggests that pandemic play could protect children’s emotional well-being. In a recent study, Thibodeau-Nielsen et al. (2021) found that child-initiated pandemic play moderated the association between caregivers’ stress and children’s emotional distress during the COVID-19 pandemic. Caregivers’ stress was positively associated with children’s emotional distress, but this was only the case for the children who engaged in pandemic play infrequently or not at all. For those who engaged in pandemic play frequently, the association between caregivers’ stress and children’s emotional distress was nonsignificant, suggesting that pandemic play protected children’s emotional well-being.

Thibodeau-Nielsen et al.’s (2021) work is informative and was one of the first studies to empirically demonstrate the potential benefits of play during the pandemic. However, because the participating families were predominantly middle- to high-income, it is unclear whether the protective role of pandemic play extends to children from low-income families. This is especially important because the pandemic exacerbated issues that low-income families were already facing, like prolonged stress, food insecurity, and access to educational experiences (Suarez-Lopez et al., 2021; Zheng et al., 2021). Indeed, the pathway from parent pandemic-related stress to child stress was intensified in low-income contexts (Kerr et al., 2021). More studies are needed to assess whether pandemic play similarly moderates the relation between caregiver stress and child emotional distress in socioeconomically disadvantaged contexts.

The goal of the present study was to replicate and extend the work of Thibodeau-Nielsen et al. (2021) by examining the association between caregivers’ stress and children’s emotional distress and the moderating role of pandemic play among low-income families. Despite evidence that pretend play, in general, serves as a protective factor against negative developmental outcomes for children in adverse contexts (Thibodeau-Nielsen et al., 2020b), it remains unclear whether playing about specific stressors, like the COVID-19 pandemic, is an effective, self-directed coping mechanism for children in high-stress, low-income contexts. We propose two hypotheses based on the research outlined above. First, we expected caregiver stress would be positively related to children’s emotional distress during the COVID-19 pandemic. Second, we hypothesized that this relation between caregiver stress and children’s emotional well-being would be moderated by pandemic play, with the association being weaker for children who engaged in pandemic play frequently.

## 2. Methods

### 2.1. Participants

We invited caregivers of 3-to-6-year-old children from Head Start preschool programs in the Midwest to complete a survey in English or Spanish, online or on paper. A total of 124 caregivers consented to participate. Five participants were excluded because they did not have a child enrolled in a Head Start preschool program or their child was too young. Another 47 were excluded due to missing data, resulting in a final sample of 72 caregivers.

Ninety percent of the final sample were mothers. Their educational level ranged from less than 12th grade (1) to graduate/professional school (7); most reported completing some college (42%). The average family income of respondents was $20,000–$30,000 annually. Children included in parent reports were, on average, 3.97 years (*SD* = 0.691), and just over half (51%) were girls. Most of the children were African American/Black (49%) and were reported to primarily speak English at home (92% of sample). Table 1 includes detailed demographic characteristics of the participants.

**Table 1:** Descriptive statistics.

	M or % of sample	SD	Range
Child age	3.97	0.691	3 to 6
Child gender	51% girls		
	49% boys		
Child race/ethnicity	49% African American/Black		
	26% European American/White		
	11% Multiracial		
	7% Hispanic/Latino(a)		
	7% Other or not reported		
Child primary language (spoken at home)	92% English		
	4% Spanish		
	4% Other or not reported		
Caregiver type	90% Mothers		
	6% Fathers		
	4% Other		
Caregiver education level	12% Less than 12^th^ grade (1)		
	18% High school graduate (2)		
	1% Some vocational school (3)		
	3% Completed vocational school (4)		
	42% Some college (5)		
	17% College graduate (6)		
	7% Graduate, professional school (7)		
Annual family income	$20,000 - $30,000	$20,000	Under $10,000 to $90,000 - $100,000
Caregiver stress (*z*-score)	0.000	0.850	−1.67 to 2.97
Child emotional distress (*z*-score)	0.000	0.913	−1.11 to 4.07
Pandemic play	68% infrequent players 32% frequent players		

### 2.2. Measures

#### 2.2.1. Caregiver stress

To assess caregiver stress, participants completed 10 items from the Perceived Stress Scale (Cohen et al., 1983; *α* = 0.758) and 4 items adapted from the Parenting Stress Scale (Berry and Jones, 1995; *α* = 0.838). These scales assess caregivers’ feelings of general stress and stress specific to caring for young children, respectively. Example items include: “How often have you felt difficulties were piling up so high that you could not overcome them?” and “How often does caring for your child(ren) take more time and energy than you have to give?” On both scales, caregivers’ responses ranged from (1) *Never* to (5) *Very Often*. We averaged items within each scale and found that the scales were positively correlated, *r*(70) = 0.445, *p* < 0.001. As such, we standardized and averaged the two scales to create a composite score of caregivers’ stress. Higher scores indicated greater stress levels.

#### 2.2.2. Child emotional distress

Consistent with the work of Thibodeau-Nielsen et al. (2021), caregivers reported on their child’s emotional distress by completing two subscales of the Child Behavior Checklist Parent-Report Form (CBCL) for ages 1.5–5 (Achenbach, 1999). One subscale consisted of 9 items on children’s emotional reactivity (*α* = 0.761) and the other included 8 items on children’s anxiety and depression (*α* = 0.745). Caregivers used a 3-point scale ranging from (0) *Not True* to (2) *Very True or Often True.* We averaged items within subscales, and given that the subscales were highly correlated, *r*(70) = 0.664, *p* < 0.001, we standardized and averaged the two subscales to create an overall score for children’s emotional distress. Higher values indicated greater emotional distress.

#### 2.2.3. Pandemic play

To assess the extent to which children engaged in pandemic-related play, caregivers were first given some brief examples of pandemic play (e.g., pretending to be a doctor treating a patient with COVID-19, pretending to be a teacher delivering lessons through a computer). Then, caregivers rated the frequency of their children’s pandemic-related play on a 7-point scale ranging from *Never* to *Multiple Times a Day*. Consistent with Thibodeau-Nielsen et al. (2021), we categorized children into two groups: those that engaged in pandemic play once a week or more were considered “frequent” players and those that engaged in pandemic play less than once a week were considered “infrequent” players.

### 2.3. Procedure

This study was approved by a university Institutional Review Board. Participants were contacted *via* advertisements to Head Start preschool programs. Interested caregivers were given an online link or a hardcopy of the survey to complete as part of a larger study. Participants were given the option to complete their surveys in English or in Spanish; most of them completed it in English (96%) and online (99%). Caregivers were instructed to answer questions based on one child and to consider “the last couple of months” in their responses. Participants who completed the survey were entered into a drawing to win one of 40 gift cards ranging in value from $25 to $200. Opportunities to participate were open from November 2020 to March 2021. During this time, there were no pandemic-related stay-at-home or lockdown restrictions in place in this region of the United States.

## 3. Results

### 3.1. Preliminary analyses

Descriptive statistics for all variables are presented in Table 1. Bivariate correlations revealed that caregiver stress was positively correlated with children’s emotional distress, *r*(70) = 0.551, *p* < 0.001. Approximately 32% of children were reported to engage in pandemic play frequently. Mean comparisons demonstrated that emotional distress levels did not vary between children who engaged in pandemic play frequently (*M* = −0.104, *SD* = 0.671) and those who engaged in pandemic play infrequently (*M* = 0.049, *SD* = 1.01), *t*(70) = 0.662, *p* = 0.510, *d* = 0.167. Also, the stress levels between caregivers of frequent players (*M* = −0.160, *SD* = 0.588) and infrequent players (*M* = 0.075, *SD* = 0.945) did not differ, *t*(64.3) = 1.286, *p* = 0.203, *d* = 0.276 (adj. for unequal variances). This suggests children’s propensity to engage in pandemic play was unrelated to children’s and caregivers’ stress levels.

Next, we tested whether children’s age, gender, race/ethnicity, primary language spoken at home, family income, and caregivers’ education level should be included as control variables by examining bivariate correlations, comparing means, and conducting chi-square analyses. Family income was negatively correlated with caregiver stress, *r*(70) = −0.257, *p* = 0.029, and children’s emotional distress, *r*(70) = −0.257, *p* = 0.029. As such, we controlled for family income in our main moderation analysis, described below (all other *p’*s ≥ 0.108).

### 3.2. Hypothesis testing

To test the association between caregivers’ stress and children’s emotional distress and examine whether pandemic play moderated that association, we used multiple regression analysis. Family income was entered on Step 1. Caregiver stress and pandemic play were entered on Step 2. Finally, an interaction term between caregiver stress and pandemic play group was calculated and entered on Step 3. The outcome variable was children’s emotional distress.

**Figure 1:**
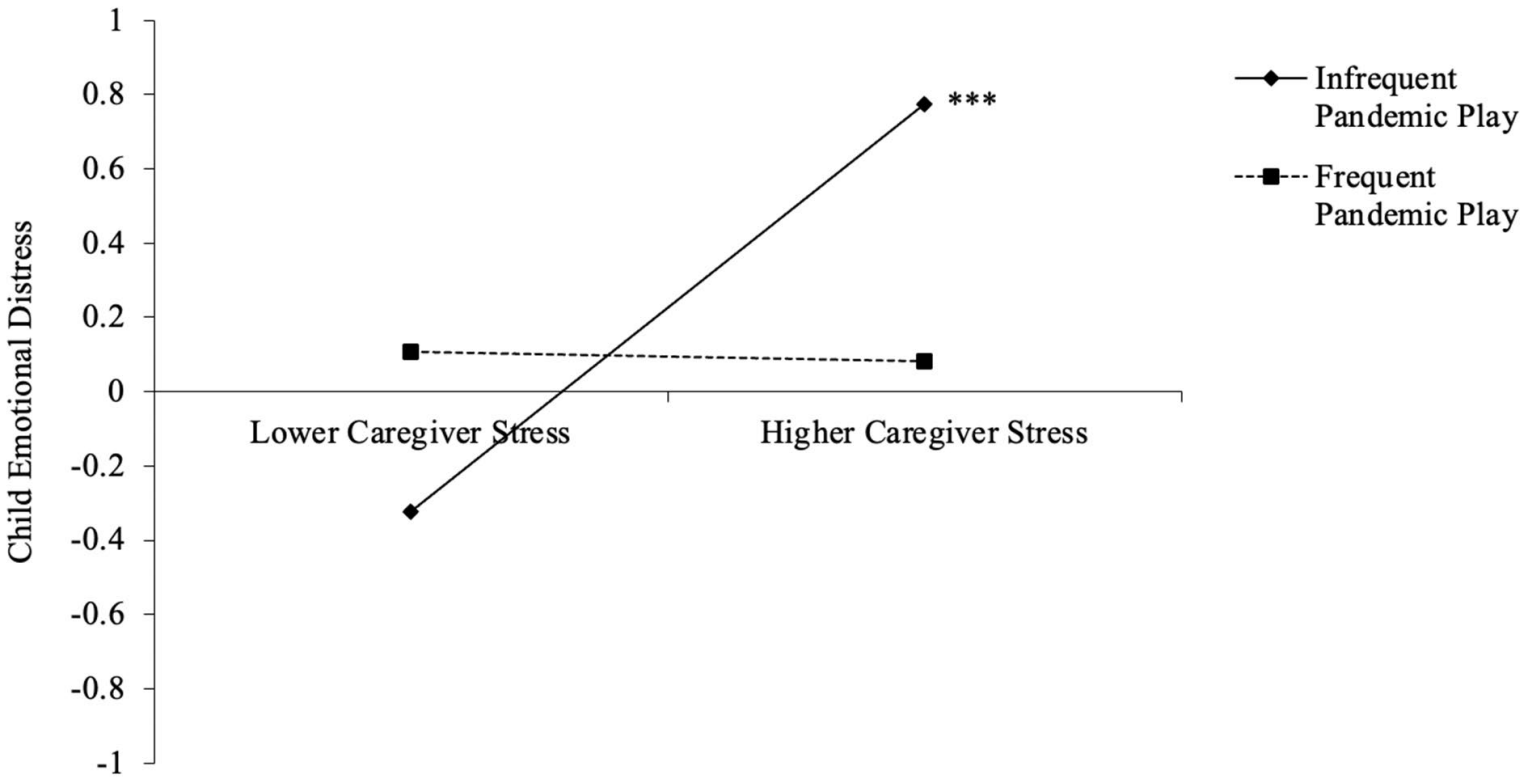
Interaction between caregivers’ stress and pandemic play on children’s emotional distress. ***p < 0.001.

Results at each step are presented in Table 2. As expected, caregiver stress was positively associated with children’s emotional distress. However, this effect was subsumed by a significant interaction effect between caregiver stress and pandemic play. The interaction effect accounted for an additional 4.7% of the variance in children’s emotional distress scores. Follow-up simple slope analyses showed that the relation between caregiver stress and child emotional distress was significant for children who engaged in pandemic play infrequently (*b* = 0.645, *SE b* = 0.118, *p* < 0.001), but not for children who engaged in pandemic play frequently (*b* = −0.016, *SE b* = 0.281, *p* = 0.954; see Figure 1). Thus, children’s frequent engagement in pandemic play appeared to weaken the association between caregivers’ stress and children’s emotional distress.

**Table 2:** Regression analysis for pandemic play moderating the association between caregiver stress and child emotional distress.

	*b*	*SE b*	*p*
Model 1, *F*(1, 70) = 4.946, *p* = 0.029, *R*^2^ = 0.066			
Constant	0.359	0.192	0.066
Family income	−0.130	0.058	0.029*
Model 2, *F*(3, 68) = 10.585, *p* < 0.001, *R*^2^ = 0.318			
Constant	0.189	0.189	0.319
Family income	−0.064	0.053	0.232
Caregiver stress	0.554	0.113	<0.001***
Pandemic play	−0.042	0.197	0.832
Model 3, *F*(4, 67) = 9.638, *p* < 0.001, *R*^2^ = 0.365			
Constant	0.225	0.184	0.226
Family income	−0.079	0.052	0.134
Caregiver stress	0.645	0.117	<0.001***
Pandemic play	−0.131	0.196	0.508
Caregiver stress x Pandemic play	−0.661	0.297	0.029*

*p ≤ 0.05, ***p ≤ 0.001.

## 4. Discussion

The goal of the present study was twofold: to examine the association between caregivers’ stress and young children’s emotional distress during the COVID-19 pandemic in a socioeconomically disadvantaged sample and to examine whether pandemic play could operate as a naturally occurring protective factor. Our results showed that caregiver stress during the pandemic was positively associated with children’s distress: as caregivers’ stress increased, so did children’s emotional distress. Other studies conducted during the pandemic have highlighted this connection as well (Spinelli et al., 2020; Thibodeau-Nielsen et al., 2021; Shelleby et al., 2022). Notably, the present study is one of the first to focus on these relations among families who regularly deal with challenges stemming from economic adversity. This represents an important contribution to the literature and our understanding of the ways in which the COVID-19 pandemic may have contributed to socioemotional vulnerability within low-income families, especially given that the cascade of negative outcomes instigated by the pandemic was heightened among families with limited financial resources (Kerr et al., 2021; Suarez-Lopez et al., 2021). Importantly, once we accounted for caregivers’ stress in our model, the association between family income and child emotional distress became nonsignificant. This finding aligns with family stress frameworks and supports research suggesting that economic hardship relates to children’s outcomes indirectly via intervening family processes, including caregiver mental health (Masarik and Conger, 2017). It is also notable that the link between caregiver and child emotional distress in the present study was stronger than in a previous study conducted with primarily middle- to high-income families using similar measures (Thibodeau-Nielsen et al., 2021). This highlights the importance of identifying protective factors, like pandemic play, that moderate the caregiver-child stress association in low-income contexts.

In this sample, caregiver stress was associated with child emotional distress for children who engaged in pandemic play infrequently but not for children who engaged in pandemic play frequently. This finding replicates and extends the work of Thibodeau-Nielsen et al. (2021) in a socioeconomically disadvantaged and ethnically diverse sample. Together, these studies suggest that child-directed play about stressful or uncertain circumstances may be a helpful coping strategy for children from a variety of backgrounds, including those from low-income contexts.

Our findings, however, raise an important question about why some children may be more inclined to engage in pandemic play than others. One possibility is that these children may simply have a higher affinity for imaginative play in general. The play literature highlights that approximately one third of children can be characterized as having a high propensity toward imaginative play (Taylor and Carlson, 1997). This is consistent with our finding that 32% of children engaged in pandemic play frequently. Children with a high propensity toward imaginative play engage in pretend play more consistently than their peers and often prefer taking on roles of other characters or pretending as if an object is something else (e.g., a blanket is a superhero cape). Perhaps children with a proclivity for imaginative play in general were more naturally drawn to cope with pandemic-related stressors and changes through imaginative, pandemic play.

As we work to further understand if and how pretend play could help children process other challenging situations (e.g., natural disasters, loss of a loved one, moving to a new school), researchers should prioritize investigating what child characteristics are associated with children’s choices to engage in pretend play about these situations. In addition to including measures of children’s propensity toward imaginative play, future studies should aim to uncover specific characteristics of pretend play that are related to positive coping. Are some themes more helpful than others? Does it matter how much agency a child has over the play scenario? How involved should adults be, and what is their role in guiding children’s play?

The findings of the present study should be viewed in light of the following limitations. First, although our findings are consistent with the idea that play could be a protective factor in the context of acute stress, because this study dealt with correlational data, we cannot draw firm conclusions about the directionality of this effect. Intervention designs will be necessary to establish causality for the potential protective effects of pretend play on child outcomes. Second, the literature would also benefit from additional longitudinal work to assess changes in children’s emotional well-being during uncertainty as they engage in pretend play over time. Third, data were collected from single informants. Researcher observations of children’s play could allow for more in-depth analyses of the features of pretend play that may be related to children’s positive coping, such as the length of the play session or level of adult involvement. Finally, the demographic characteristics of our sample may not fully capture the range of experiences of children growing up in contexts of economic adversity. Additional research should explore the utility of pretend play as a potential protective factor in additional samples of children from diverse backgrounds.

Despite the need for more research, the current findings corroborate decades of research highlighting the potential value of pretend play in early development (e.g., Taylor and Carlson, 1997; Russ, 2007; Karniol et al., 2011; White and Carlson, 2016; Goldstein and Lerner, 2018; Thibodeau-Nielsen et al., 2020a; White et al., 2021; Hollenstein et al., 2022). Together, they suggest several practical implications. Outside of a pandemic context, families with few financial resources are more likely than their affluent counterparts to face significant life transitions, like housing insecurity or job loss (Laughlin, 2014). The present study suggests that playing through major life disruptions may be a potentially powerful strategy to promote resiliency for children facing economic adversity. Importantly, almost all children have a natural capacity to pretend (Eibl-Eibesfeldt, 1989; Lillard, 2017), meaning that it typically does not require specialized training or intensive guidance from an adult. Furthermore, because pretend play is not bound by reality, everyday inexpensive items can be used to facilitate this type of play. For example, a box can be used as a rocket ship in everyday pretend play, and a straw can be used as a thermometer in pretend play related to the pandemic. These features of pretend play make it an accessible tool that builds on children’s existing abilities and strengths to promote well-being, suggesting it could be an equitable point of intervention for children from a variety of economic contexts.

### 4.1. Conclusion

The current study is consistent with previous findings that caregiver stress is related to heightened child emotional distress during the pandemic (e.g., Spinelli et al., 2020; Thibodeau-Nielsen et al., 2021); it is one of the first to study this association in a primarily lower-income sample. Importantly, we found that the relation between caregiver stress and children’s emotional distress depended on children’s engagement in pandemic play, with the relationship being nonsignificant when children were reported to engage in pandemic play frequently. Although more research is needed to understand the specific characteristics of children that choose to engage in hardship-related play and whether this play contributes to children’s resiliency, our findings add to a growing literature suggesting the importance of child-directed play in early development (e.g., Hirsh-Pasek et al., 2009; Weisberg et al., 2013; Yogman et al., 2018; Zosh et al., 2018).

## Data availability statement

Deidentified data supporting the conclusions of this article will be made available by the authors upon request.

## Ethics statement

This study involving human participants was reviewed and approved by University of Missouri Institutional Review Board. The participants provided their written informed consent to participate in this study.

## Author contributions

MR-P conducted analyses, drafted the initial manuscript, reviewed, and revised the manuscript. RT-N contributed to the conception and design of the study, collected data, conducted analyses, assisted in drafting of the initial manuscript, reviewed, and revised the manuscript. SD contributed to the design of the study, drafted the initial manuscript, collected data, reviewed, and revised the manuscript. AW-D contributed to the design of the study, collected data, reviewed, and revised manuscript. FP and RW contributed to the conception and design of the study, collected data, reviewed, and revised the manuscript. CC conducted analyses, reviewed, and revised the manuscript. All authors approved the final manuscript as submitted and agree to be accountable for all aspects of the work.

## Funding

This study was partially funded by the College of Human Environmental Sciences and Department of Human Development and Family Science at the University of Missouri. This material is based upon work supported by the National Science Foundation Graduate Research Fellowship Program under Grant No. 1842485, awarded to AW-D. Any opinions, findings, and conclusions or recommendations expressed in this material are those of the author(s) and do not necessarily reflect the views of the National Science Foundation.

## Conflict of interest

The authors declare that the research was conducted in the absence of any commercial or financial relationships that could be construed as a potential conflict of interest.

## Publisher’s note

All claims expressed in this article are solely those of the authors and do not necessarily represent those of their affiliated organizations, or those of the publisher, the editors and the reviewers. Any product that may be evaluated in this article, or claim that may be made by its manufacturer, is not guaranteed or endorsed by the publisher.
